# Maternal Obesity and Developmental Programming of Metabolic Disorders in Offspring: Evidence from Animal Models

**DOI:** 10.1155/2011/592408

**Published:** 2011-09-28

**Authors:** M. Li, D. M. Sloboda, M. H. Vickers

**Affiliations:** Liggins Institute and the National Research Centre for Growth and Development, University of Auckland, Auckland 1142, New Zealand

## Abstract

The incidence of obesity and overweight has reached epidemic proportions in the developed world as well as in those countries transitioning to first world economies, and this represents a major global health problem. Concern is rising over the rapid increases in childhood obesity and metabolic disease that will translate into later adult obesity. Although an obesogenic nutritional environment and increasingly sedentary lifestyle contribute to our risk of developing obesity, a growing body of evidence links early life nutritional adversity to the development of long-term metabolic disorders. In particular, the increasing prevalence of maternal obesity and excess maternal weight gain has been associated with a heightened risk of obesity development in offspring in addition to an increased risk of pregnancy-related complications. The mechanisms that link maternal obesity to obesity in offspring and the level of gene-environment interactions are not well understood, but the early life environment may represent a critical window for which intervention strategies could be developed to curb the current obesity epidemic. This paper will discuss the various animal models of maternal overnutrition and their importance in our understanding of the mechanisms underlying altered obesity risk in offspring.

## 1. Background

The current epidemic of obesity and related metabolic disorders has been seen as a symptom of affluence with the primary cause relating to the development of an obesogenic environment and ease of access to highly calorific foods and reduced energy expenditure in work and leisure activities [1]. The metabolic syndrome is characterised by the clustering of cardiovascular risk factors including diabetes, obesity, hyperlipidaemia, and hypertension and is likely the result of complex interactions between genes, dietary intake, physical activity, and the environment. Within the cluster of risk traits for the metabolic syndrome, insulin resistance and visceral obesity have been recognized as the most important causal factors [2]. A number of genes have been identified that are associated with obesity and metabolic syndrome in humans [1, 3], but the genetic component of this condition cannot account for the marked increases in the prevalence of obesity and metabolic syndrome in recent years. In this context, the developmental origins of health and disease (DOHaD) hypothesis has highlighted the link between the periconceptual, fetal, and early infant phases of life and subsequent development of adult obesity and the metabolic syndrome [4–6].

The mechanisms underpinning the developmental programming framework and the role of genetic versus environmental factors remain speculative. One general thesis is that in response to an adverse intrauterine environment the fetus adapts its physiological development to maximize its immediate chances for survival. These adaptations may include resetting metabolic homeostasis set points, endocrine systems, and downregulating of growth, commonly manifest in an altered birth phenotype. More recently the “predictive adaptive response” (PARs) hypothesis proposes that the degree of mismatch between the pre- and postnatal environments is a major determinant of subsequent disease risk [7, 8]. Thus, it is thought that whilst adaptive changes in fetal physiology may be beneficial for short-term survival *in utero,* they may be maladaptive in later life, contributing to adverse health outcomes when offspring are exposed to catch-up growth, diet-induced obesity, and other factors [8, 9]. 

Animal models have been extensively used to study the basic physiological principles underlying the DOHaD hypothesis and are essential to the search for the mechanistic links between prenatal and postnatal influences and risk for developing the metabolic syndrome in later life. Epidemiological data suggest that developmental programming occurs within the normal range of birth size [10, 11], but most early experimental work has focused on fetal growth restriction in the assumption that insults impairing fetal growth are likely to be those triggering developmental programming. However, over recent years there has been an increasing focus on developing models of maternal obesity. Obesity in pregnancy and gestational diabetes represent a special problem, not only as a result of their immediate adverse effects on maternal health and pregnancy outcome, but also because of growing evidence for their persistent and deleterious effects on the developing offspring [12]. However, in obese women, it is difficult to discern between genetic and environmental contributions in offspring disease risk. Several obesogenic animal models, primarily performed in the rodent, show a relatively common phenotype of metabolic disorders in offspring, but the magnitude of effects differs with the timing of the nutritional challenge and diet composition [13]. A recent systematic review by Ainge et al. showed that although a maternal HF diet was associated with a real risk of type 2 diabetes and obesity in male offspring, inconsistencies between the studies limited an examination of the *mechanisms* underlying phenotype development [14]. This paper will provide a current summary of animal models of maternal obesity including model species, nature and timing of dietary manipulations, phenotypic outcomes in offspring, possible mechanisms, and the potential role of epigenetics.

## 2. Animal Models of Maternal Obesity

### 2.1. Rodent Models

**Figure 1:**
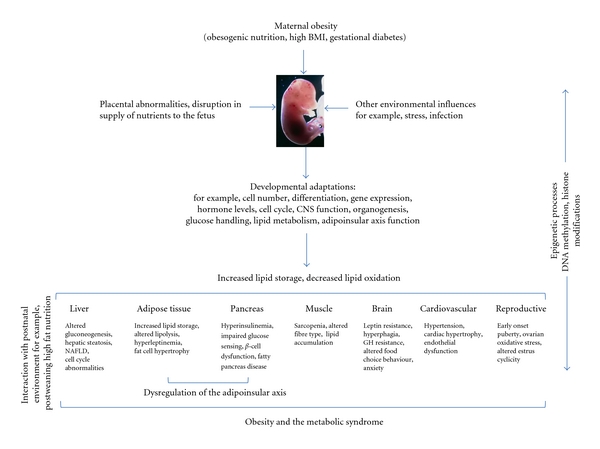
Basic schematic outlining consequences of a maternal obesogenic environment on the health and well-being of offspring. These effects can be modified by the timing and duration of exposure to the obesogenic environment as well as gender and composition of the HF diet (e.g., fat source). Programming effects can be further exacerbated in the presence of a suboptimal postweaning nutritional environment.

**Figure 2:**
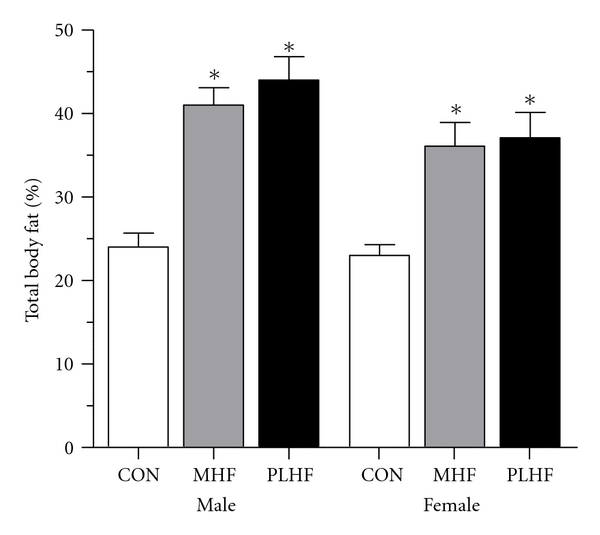
Total body fat mass (%) as quantified by dual energy X-ray absorptiometry (DEXA) in adult (day 150) male and female rat offspring of HF-fed mothers. CON = offspring of control pregnancies, MHF = offspring of mothers fed an HF diet from weaning and throughout pregnancy and lactation, PLHF mothers fed a control diet until conception, and an HF diet throughout pregnancy and lactation. Note that a preconceptional HF diet did not confer an altered risk for obesity development over that of HF exposure during pregnancy and lactation alone. Data are means ± SEM, *n* = 10-11 per group, *P < 0.05 versus CON. Modified from Howie et al. [29].

The rodent is the most commonly used model species for investigation of developmental programming via a maternal obesogenic nutritional environment. A maternal cafeteria or high-fat (HF) diet has been shown to induce obesity, insulin and leptin resistance [15–17], hypertension [18–21], fatty pancreas disease [22], hepatic steatosis, and nonalcoholic fatty liver disease in offspring [23–26] (Figure 1). It has also been reported that maternal adiposity, and not dietary fat *per se*, induces hyperleptinemia and insulin resistance in offspring, as well as an increased body weight that persists into adulthood [27]. Even mild maternal overnutrition has been shown to induce increased adiposity, glucose intolerance, and altered brain appetite regulators in offspring [28]. Our own previous work has shown that a moderate maternal HF diet results in significant obesity and hyperinsulinemia in male and female offspring, independent of the level of preconceptional obesity [29] (Figure 2).

In general, two main approaches have been utilised, a high-fat and/or high-sugar purified diet approach or a “cafeteria diet” designed to mimic a complex western style diet [17, 30, 31]. Both approaches have been extensively utilised over recent years and have provided important insights into disease development, particularly in relation to the development of the metabolic syndrome. There has been some recent debate as to which dietary approach is more closely aligned to the human setting. A purified HF diet normally utilises a modification of a single fat source, for example, lard, in order to induce excess weight gain. Use of these purified “open source” diets has the benefit for targeted mechanistic studies in that manipulation of a single dietary component can be easily undertaken but has the downside that rodents are able to regulate total caloric intake when fed a standard HF diet [32, 33]. A cafeteria diet, a mix of foods typified in the human setting such as highly processed snack food, mimics a more western style diet. However, interpretation of specific macronutrient effects is very difficult due to the widely varied macronutrient sources across the added foods and the dietary interaction across the varied fat, protein, and carbohydrate backgrounds. Further, there is some evidence that specific components utilised in the cafeteria diet may have deleterious effects such as those related to dairy intake in the rodent [34, 35] and oxidative stress [36]. Recent work by Sampey et al. investigated the obesogenic and inflammatory consequences of a cafeteria diet compared to a lard-based 45% (of calories) HF diet in the rodent. Both diets resulted in increased adiposity and hepatosteatosis but cafeteria-fed rats displayed increased inflammation in white fat, brown fat, and liver compared to HF and control groups [37]. Interestingly, the review by Ainge et al. showed that a maternal HF diet did confer an enhanced risk for T2DM and obesity; however, poor glycaemic control across the multiple studies examined appeared to be independent of maternal obesity, birth weight, and level of postweaning diet [14].

### 2.2. Sheep Models

Sheep models are less studied than the rodent, but there is strong evidence from ovine models that maternal obesity predisposes to altered growth and metabolic sequelae in offspring, data that closely parallels that observed in the small animal models. In a study by Zhang et al., an ovine model of maternal obesity was utilised in which ewes were overfed in order to induce obesity at conception and throughout gestation. At mid-gestation, fetuses from obese ewes were macrosomic, hyperglycemic, and hyperinsulinemic and exhibited markedly increased pancreatic weight and *β*-cell numbers compared with fetuses of ewes fed to requirements. These data also demonstrated differential impacts of maternal obesity on fetal pancreatic growth and *β*-cell numbers during early and late gestation. During the first half of gestation there was a marked increase in pancreatic growth, *β*-cell proliferation, and insulin secretion, followed by a reduction in pancreatic growth and *β*-cell numbers in late gestation, resulting in reduced circulating insulin at term [38].

Maternal obesity and increased nutrient intake before and during gestation in the ewe is known to result in altered growth, adiposity, and glucose tolerance in adult offspring [39]. As with the rodent studies, different levels of overnutrition and weight gain during pregnancy have differential effects on fetal growth and organ development [40]. Maternal overnutrition in late pregnancy results in an upregulation of PPAR*γ*-activated genes in fetal visceral fat and a subsequent increase in the mass of subcutaneous fat in the postnatal lamb. Exposure to maternal overnutrition during the periconceptional period alone, however, was shown to result in an increase in total body fat mass only in female lambs with a dominant effect on visceral fat depots. Therefore, it was proposed that the early programming of later obesity may result from “two hits”, the first occurring as a result of maternal overnutrition during the periconceptional period and the second occurring as a result of increased fetal nutrition in late pregnancy [40].

In contrast to the rodent literature, where maternal obesity has been shown to result in an amplified and prolonged neonatal leptin surge [41], data in the sheep has shown that maternal obesity eliminates the neonatal lamb plasma leptin peak [39]. These differences may be explained via the relative immaturity of the rat at birth compared to the lamb, with the newborn lamb being born at a more advanced level of maturity equivalent to humans [39].

### 2.3. Nonhuman Primate

The extensive body of evidence in small animal models and those undertaken in the sheep linking a maternal HF diet to disease risk has been supported by studies, albeit limited, in the nonhuman primate. One of the earliest, in the baboon, showed that overfeeding in the preweaning period permanently increased adiposity in offspring through fat cell hypertrophy, a gender-dependent effect in the females only [42]. Maternal HF diet triggers lipotoxicity in the fetal liver of macaques [43] and predisposes the offspring to develop nonalcoholic fatty liver disease in adulthood. This aligns closely with work in the rodent where a maternal high-fat diet has been shown to lead to “developmental priming” of hepatic steatosis in offspring [24]. Data by Farley and colleagues demonstrated that for normal weight offspring of obese baboons, placental, and fetal phenotypes were consistent with those described for large-for-gestational age human fetuses [44]. Recent work in the macaque by Grayson et al. showed that offspring of mothers fed an HF diet preconceptionally and throughout pregnancy had smaller body weights in the early third trimester but displayed catch-up growth and increased adiposity in the postnatal period—the offspring also developed early-onset excess weight gain independent of postnatal diet [45]. A primary finding of this work was the effect of a maternal HF diet on the offspring's melanocortin system. Third-trimester fetuses from mothers on HF showed increases in proopiomelanocortin mRNA expression, whereas agouti-related protein mRNA and peptide levels were decreased in comparison with control fetuses. In this study, a subgroup of adult HF animals was switched to a control diet during pregnancy (diet reversal). Although at the time of conception the diet reversal animals remained significantly obese and insulin resistant compared to controls, the offspring displayed normal melanocortin levels. These data suggest that chronic consumption of an HF diet during pregnancy, *independent *of maternal obesity and diabetes, can lead to widespread activation of proinflammatory cytokines that may alter the development of the melanocortin system. This aligns with the work in the rat by Howie et al., whereby preconceptional maternal obesity did not impact on obesity in offspring above that induced by an HF diet during pregnancy and lactation alone [29]. It has also recently been shown that chronic consumption of an HF diet during pregnancy causes perturbations in the serotonergic system and increased anxiety-like behavior in nonhuman primate offspring [46].

In the Japanese macaque, consumption of an HF diet, independent of maternal obesity, increased placental inflammatory cytokines and the expression of Toll-like receptor 4 [47]. HF diet consumption also reduced volume blood flow on the fetal side of the placenta and significantly increased the frequency of both placental infarctions and stillbirth. These results suggest that an HF diet, independent of obesity, decreases uterine volume blood flow with maternal obesity and insulin resistance further exacerbating placental dysfunction and resulting in an increased frequency of stillbirth [47]. This aligns with the rodent data whereby a maternal HF diet has been shown to result in reduced fetal and placental junctional zone weights [48].

## 3. Mechanisms

The mechanisms underpinning maternal obesity and programming of obesity risk in offspring are not well defined. Limited data to date highlight the role of altered leptin production and regulation and changes in the hypothalamic regulation of key genes involved in appetite control and energy balance. There is also evidence of altered skeletal muscle metabolism and maternal HF diet-induced effects on placental structure and function.

### 3.1. Leptin and the Regulation of Energy Balance

One of the most studied and consistent observations is hyperphagia and altered energy intake [49]. Early data suggested a change in food preference with maternal HF offspring displaying a preference for junk food over chow [50] and more recent studies demonstrate hyperphagia in chow-fed offspring of obese mothers [41, 51]. Programmed resistance to the adipokine leptin is as a prime candidate for the mechanism predisposing towards an altered energy balance. The leptin surge has been well characterised in the rodent; it appears to be neonatal in origin and is associated with an upregulation in leptin mRNA expression in adipose tissue over the same time course [41, 52]. Maternal obesity has been shown to result in an amplified and prolonged leptin surge in neonatal rat offspring [41]. In the rat, the leptin surge is seen as a consequence of elevated maternal serum leptin during the early postnatal period leading to elevated milk leptin concentrations and hyperleptinemia in suckling offspring. However, there are some inconsistencies across studies—milk from HF dams has been shown to have significantly higher fat content compared to controls associated with increased insulin but not leptin concentrations [53]. It must be noted, however, that although the leptin surge in the rodent has been well described, the precise timing and characteristics of the neonatal leptin peak have not been well defined in offspring of either normal or obese mothers in any precocial species. Further studies have described lactational failure and increased neonatal mortality in offspring of HF fed mothers [54]. It has also recently been reported that obese mothers spend significantly more time nursing their young which could manifest as programming changes in the HPA via altered maternal care as described by Meaney and colleagues [55, 56].

### 3.2. Hypothalamic Reprogramming

Despite the increasing evidence for a neurotropic role of leptin in the rodent, the potential role in humans and the timing of the possible leptin surge is less defined. Kirk et al. have shown that maternal diet-induced obesity permanently influences central processes regulating food intake in offspring via programming of leptin resistance and altered hypothalamic functions involving the arcuate nucleus and paraventricular nucleus. Further, intrauterine and early postnatal overnutrition programmes hypothalamic neurons expressing the appetite stimulator neuropeptide Y (NPY) and suppressor proopiomelanocortin (POMC) in offspring at weaning [15]. However, the long-term effects of such programming and its interactions with postweaning HF diet consumption remain unclear. Several studies have highlighted alterations in peroxisome proliferator activated receptor (PPAR) gene expression in offspring of obese mothers, which may contribute to the disturbed lipid homeostasis. HF offspring have decreased hepatic PPAR*γ* expression compared with controls and reduced hepatic PPAR*α* expression which negatively correlated with serum triglyceride levels [57].

### 3.3. Skeletal Muscle and Locomotor Activity

Work by Simar et al. in the adult rat revealed an interaction between maternal obesity and postnatal overnutrition on skeletal muscle metabolism, a postweaning HF diet exerted an additive effect to that of maternal obesity on body weight and skeletal muscle markers of glucose and lipid metabolism but not on plasma glucose and insulin levels, suggesting that maternal obesity and postnatal overnutrition impair skeletal muscle function via different mechanisms [58]. Reduced muscle mass has also been reported in 3- and 6-month-old male and female offspring from obese mice [31] and reduced muscle force in offspring of mothers fed a junk food diet [59]. Work in the sheep matches closely that reported for the rat; lambs born to obese mothers have impaired insulin signalling in muscle compared with control lambs which correlated with increased intramuscular triglycerides and higher expression of fatty acid transporters and PPAR-*γ* [60].

Although several studies have examined changes in locomotor activity in the setting of maternal undernutrition [61–63], data on energy expenditure and physical activity in models of maternal obesity have yet to be undertaken despite the numerous studies that have observed differences in muscle development in offspring of obese mothers.

### 3.4. Placental Function

Altered placental function in the setting of maternal obesity has also been the focus of several investigations. Our group and others have reported altered placental structure and function as a result of a maternal HF diet across a range of experimental models. In the pregnant rat, a maternal HF diet has been shown to reduce growth of the fetus and the placental junctional zone, but not placental labyrinth zone growth [48]. In the pregnant sheep, maternal obesity markedly increases placental fatty acid transporter expression and inflammatory signalling pathways and enhances cytokine expression in mid-gestation [57]. Similarly, maternal obesity in the baboon is associated with a maternal inflammatory state and induces structural and functional changes in the placenta [44].

### 3.5. Interventions

Until relatively recently, developmental programming was seen as an irreversible change in developmental trajectory. Outside of the early taurine reversal work in the setting of the maternal low protein model [64], there is a paucity of data on intervention strategies whether it be nutritional or targeted pharmacologic approaches. It has recently been shown that interventions with leptin, folic acid, and exendin-4 in the early phases of developmental plasticity can ameliorate or reverse some of the effects associated with developmental programming [65–67]. However, these agents were examined in the context of maternal nutritional deprivation and have not been studied in the setting of maternal overnutrition, despite the commonality of offspring phenotypes across the disparate nutritional models. Similarly, exercise has been shown to have beneficial effects in obesity-prone offspring of undernourished mothers [62, 68], but no studies to date have examined exercise interventions in offspring of HF-fed mothers.

There is evidence for a role of diet reversal in ameliorating the effects of maternal obesity on offspring outcome. In the nonhuman primate, diet reversal from an HF to control diet during pregnancy led to normalisation of the melanocortin levels, improvements in fetal hepatic triglycerides, and partial normalization of the expression of gluconeogenic enzymes. These results suggested that simply changing to a normal low-fat diet, specifically during pregnancy, can lower, but not eliminate, the risk for fetal hepatic steatosis [43]. Similar results have been shown in the rat whereby dietary intervention prior to pregnancy reversed metabolic programming in male offspring of obese rats [69].

## 4. Role of Epigenetics

Epigenetic processes lead to heritable changes in gene function by altering DNA chemistry independent of sequence and may be responsible for tissue-specific gene expression during differentiation. Epigenetic modifications may be one mechanism by which exposure to an altered intrauterine milieu or metabolic perturbation may influence the phenotype of the organism much later in life [70]. However, how the four epigenetic modalities—DNA methylation, non-coding RNA, transcription factors, and histone modifications—contribute to epigenetic memory and how epigenomic changes may mediate the altered control of fetal gene expression as a consequence of maternal obesity are not well characterised. 

Experimental data in rodents and recent observations in humans suggest that epigenetic changes in regulatory and growth-related genes play a significant role in mediating the pathophysiological phenotypes derived from developmental programming [71, 72]. Histone modifications in conjunction with DNA methylation regulate chromatin structure and gene expression. However, it is still debated where early life and/or environmental factors can influence the “histone” code in a manner similar to their influence on DNA methylation [73]. 

Adversity during pregnancy or early neonatal life in experimental programming models results in changes in promoter methylation, therefore, directly or indirectly, affect gene expression in pathways associated with a range of physiologic processes [74]. For example, in the rat, altered promoter methylation and downstream changes in gene expression have been shown for the hepatic glucocorticoid receptor (GR) and PPAR-*α* [66, 75], influencing carbohydrate and lipid metabolism. The phenotypic effects of epigenetic modifications during development may not manifest until later in life, especially if they affect genes modulating responses to later environmental challenges, such as dietary challenges with an HF diet. The timing of the developmental windows and the induction of epigenetic changes in key physiologic systems is not well characterised, but it appears to extend from the periconceptional period [76] into postnatal life [77, 78]. Many of the genes regulated by epigenetic change do not appear to be classically imprinted (expressed according to the parental origin of the allele), although some imprinted genes may show altered expression after perturbations during early development, such as if blastocyst culture *in vitro *is prolonged [79].

It has been shown that the promoter in the leptin gene is subject to epigenetic programming, and leptin gene expression can be modulated by DNA methylation [80–82]. Recent studies report that impaired glucose tolerance during pregnancy is associated with adaptations in leptin gene DNA methylation although the functional significance of these changes is not yet clear [83]. Yokomori et al. demonstrated that methylation of specific CpG sites and a methylation-sensitive protein could contribute to changes in leptin gene expression during adipocyte differentiation in 3T3-L1 cells [84]. In addition, differential DNA methylation was observed in promoters of genes involved in glucose metabolism including GLUT4 [85] and uncoupling protein (UCP)-2 [86], both major contributors to the development of T2DM.

Developmental epigenetics is believed to establish “adaptive phenotypes” to meet the demands of the later-life environment [73, 87]. Implicit in this concept is an important process of causality on the cellular level, regulating growth and tissue differentiation and involving chemical changes to the DNA or associated proteins. Once the mechanistic basis of the disease is understood, epigenetic processes are potentially reversible and intervention and strategies aimed at reversal could be devised and implemented. A recent study suggested that a substantial component of metabolic disease risk has a prenatal developmental basis and that perinatal epigenetic analysis may have utility in identifying individual vulnerability to later obesity and metabolic disease [88]. In this study, they reported a link between gene promoter methylation of retinoid x-receptor alpha (RXRA) in umbilical cord tissue and later risk of childhood adiposity.

## 5. Paternal Transmission

There is now evidence supporting a role for paternal transmission of disease risk across generations. Ng et al. recently reported that a paternal high-fat diet can program *β*-cell “dysfunction” in rat F1 female offspring. Paternal HF diet altered the expression of 642 pancreatic islet genes in adult female offspring including functional gene clusters related to cation and ATP binding, cytoskeleton, and intracellular transport. Broader pathway analysis demonstrated involvement of calcium-, MAPK-, and Wnt-signalling pathways, apoptosis, and cell cycle regulation. Hypomethylation of the Il13ra2 gene, which showed the highest fold difference in expression, was demonstrated. This work provided the first evidence in mammals of nongenetic, intergenerational transmission of metabolic sequelae of an HFD from father to offspring [89]. Yazbek et al. also provided evidence for paternal transgenerational genetic effects on body weight and food intake. Utilising the obesity-resistant 6C2d congenic strain, which carries the Obrq2aA/J allele on an otherwise C57BL/6J background, obesity-resistant and hypophagic phenotypes were transmitted through the paternal lineage but not the maternal lineage with equal strength for at least two generations [90]. Of note, a recent study has shown that a maternal HF diet has effects on third-generation female body size via the paternal lineage [91] which supports a stable germline-based transgenerational mode of inheritance. The extent of the contribution of obese fathers on offspring phenotype development is now an increasing area of research, particularly as regards the role of nongenetic factors in the causal pathway.

## 6. Summary

Animal studies across a range of species and varied obesogenic diets have provided clear evidence linking maternal obesity and increased risk for obesity in offspring and are invaluable tools in the investigation of mechanisms underpinning this linkage. Given the current obesity epidemic and the increasing number of obese women entering pregnancy, there is an urgent need for interventional strategies that target developmentally programmed obesity. The evidence available on short- and long-term health impact for mother and child currently favours actions directed at controlling prepregnancy weight and preventing obesity in females of reproductive ages [92] although more trials are needed to evaluate the effects of nutritional and behavioural interventions in pregnancy outcomes. Moreover, suggestions that maternal obesity may transfer obesity risk to child through non-Mendelian (e.g., epigenetic) mechanisms require more long-term investigation. Of note, evidence for the programming of obesity and several other features of the metabolic syndrome have been observed under both nutrient restriction (caloric, protein, iron) and overnutrition studies, possibly suggestive of a commonality of mechanism. The perinatal environment provides a potential therapeutic target, and focusing on this specific developmental stage may translate into improved interventional strategies to stem the growing epidemic of obesity. Failure to recognize that maternal diet and maternal obesity play a critical role in developmental programming of adult disease may ultimately accelerate the obesity epidemic through successive generations, independent of further genetic or environmental factors.

## References

[1] Bell CG, Walley AJ, Froguel P (2005). The genetics of human obesity. *Nature Reviews Genetics*.

[2] Duvnjak L, Duvnjak M (2009). The metabolic syndrome—an ongoing story. *Journal of Physiology and Pharmacology*.

[3] Hofbauer KG (2002). Molecular pathways to obesity. *International Journal of Obesity and Related Metabolic Disorders*.

[4] Godfrey KM, Barker DJP (2000). Fetal nutrition and adult disease. *The American Journal of Clinical Nutrition*.

[5] Breier BH, Vickers MH, Ikenasio BA, Chan KY, Wong WPS (2001). Fetal programming of appetite and obesity. *Molecular and Cellular Endocrinology*.

[6] Ravelli AC, van Der Meulen JH, Osmond C, Barker DJ, Bleker OP (1999). Obesity at the age of 50 y in men and women exposed to famine prenatally. *The American Journal of Clinical Nutrition*.

[7] Gluckman PD, Hanson MA (2004). Developmental origins of disease paradigm: a mechanistic and evolutionary perspective. *Pediatric Research*.

[8] Gluckman PD, Hanson MA, Beedle AS, Spencer HG (2008). Predictive adaptive responses in perspective. *Trends in Endocrinology and Metabolism*.

[9] Gluckman PD, Hanson MA (2004). Living with the past: evolution, development, and patterns of disease. *Science*.

[10] Barker DJ (2007). Obesity and early life. *Obesity Reviews*.

[11] Barker DJP (2007). The origins of the developmental origins theory. *Journal of Internal Medicine*.

[12] Nathanielsz PW, Poston L, Taylor PD (2007). In utero exposure to maternal obesity and diabetes: animal models that identify and characterize implications for future health. *Obstetrics and Gynecology Clinics of North America*.

[13] Armitage JA, Lakasing L, Taylor PD (2005). Developmental programming of aortic and renal structure in offspring of rats fed fat-rich diets in pregnancy. *Journal of Physiology*.

[14] Ainge H, Thompson C, Ozanne SE, Rooney KB (2010). A systematic review on animal models of maternal high fat feeding and offspring glycaemic control. *International Journal of Obesity*.

[15] Morris MJ, Chen H (2009). Established maternal obesity in the rat reprograms hypothalamic appetite regulators and leptin signaling at birth. *International Journal of Obesity*.

[16] Taylor PD, Poston L (2007). Developmental programming of obesity in mammals. *Experimental Physiology*.

[17] Bayol SA, Simbi BH, Stickland NC (2005). A maternal cafeteria diet during gestation and lactation promotes adiposity and impairs skeletal muscle development and metabolism in rat offspring at weaning. *Journal of Physiology*.

[18] Liang C, Oest ME, Prater MR (2009). Intrauterine exposure to high saturated fat diet elevates risk of adult-onset chronic diseases in C57BL/6 mice. *Birth Defects Research Part B*.

[19] Elahi MM, Cagampang FR, Mukhtar D, Anthony FW, Ohri SK, Hanson MA (2009). Long-term maternal high-fat feeding from weaning through pregnancy and lactation predisposes offspring to hypertension, raised plasma lipids and fatty liver in mice. *The British Journal of Nutrition*.

[20] Khan I, Dekou V, Hanson M, Poston L, Taylor P (2004). Predictive adaptive responses to maternal high-fat diet prevent endothelial dysfunction but not hypertension in adult rat offspring. *Circulation*.

[21] Samuelsson AM, Morris A, Igosheva N (2010). Evidence for sympathetic origins of hypertension in juvenile offspring of obese rats. *Hypertension*.

[22] Oben JA, Patel T, Mouralidarane A (2010). Maternal obesity programmes offspring development of non-alcoholic fatty pancreas disease. *Biochemical and Biophysical Research Communications*.

[23] Bouanane S, Merzouk H, Benkalfat NB (2010). Hepatic and very low-density lipoprotein fatty acids in obese offspring of overfed dams. *Metabolism*.

[24] Bruce KD, Cagampang FR, Argenton M (2009). Maternal high-fat feeding primes steatohepatitis in adult mice offspring, involving mitochondrial dysfunction and altered lipogenesis gene expression. *Hepatology*.

[25] Gregorio BM, Souza-Mello V, Carvalho JJ, Mandarim-de-Lacerda CA, Aguila MB (2010). Maternal high-fat intake predisposes nonalcoholic fatty liver disease in C57BL/6 offspring. *The American Journal of Obstetrics and Gynecology*.

[26] Oben JA, Mouralidarane A, Samuelsson AM (2010). Maternal obesity during pregnancy and lactation programs the development of offspring non-alcoholic fatty liver disease in mice. *Journal of Hepatology*.

[27] White CL, Purpera MN, Morrison CD (2009). Maternal obesity is necessary for programming effect of high-fat diet on offspring. *The American Journal of Physiology—Regulatory Integrative and Comparative Physiology*.

[28] Rajia S, Chen H, Morris MJ (2010). Maternal overnutrition impacts offspring adiposity and brain appetite markers-modulation by postweaning diet. *Journal of Neuroendocrinology*.

[29] Howie GJ, Sloboda DM, Kamal T, Vickers MH (2009). Maternal nutritional history predicts obesity in adult offspring independent of postnatal diet. *Journal of Physiology*.

[30] Ong ZY, Muhlhausler BS (2011). Maternal “junk-food” feeding of rat dams alters food choices and development of the mesolimbic reward pathway in the offspring. *The FASEB Journal*.

[31] Samuelsson AM, Matthews PA, Argenton M (2008). Diet-induced obesity in female mice leads to offspring hyperphagia, adiposity, hypertension, and insulin resistance: a novel murine model of developmental programming. *Hypertension*.

[32] Guo F, Jen KL (1995). High-fat feeding during pregnancy and lactation affects offspring metabolism in rats. *Physiology and Behavior*.

[33] Taylor PD, Khan IY, Lakasing L (2003). Uterine artery function in pregnant rats fed a diet supplemented with animal lard. *Experimental Physiology*.

[34] Nielsen TS, Purup S, Wärri A, Godschalk RW, Hilakivi-Clarke L (2011). Effects of maternal exposure to cow's milk high or low in isoflavones on carcinogen-induced mammary tumorigenesis among rat offspring. *Cancer Prevention Research*.

[35] Zhou H, Qin LQ, Tang FL, Ma DF, Wang PY, Wang Y (2007). Effect of milk on the 7,12-dimethylbenz[a]-anthracene-induced mammary tumor model in rat. *Food and Chemical Toxicology*.

[36] Diniz YS, Fernandes AA, Campos KE, Mani F, Ribas BO, Novelli EL (2004). Toxicity of hypercaloric diet and monosodium glutamate: oxidative stress and metabolic shifting in hepatic tissue. *Food and Chemical Toxicology*.

[37] Sampey BP, Vanhoose AM, Winfield HM (2011). Cafeteria diet is a robust model of human metabolic syndrome with liver and adipose inflammation: comparison to high-fat diet. *Obesity*.

[38] Zhang L, Long NM, Hein SM, Ma Y, Nathanielsz PW, Ford SP (2011). Maternal obesity in ewes results in reduced fetal pancreatic beta-cell numbers in late gestation and decreased circulating insulin concentration at term. *Domestic Animal Endocrinology*.

[39] Long NM, Ford SP, Nathanielsz PW (2011). Maternal obesity eliminates the neonatal lamb plasma leptin peak. *Journal of Physiology*.

[40] George LA, Uthlaut AB, Long NM (2010). Different levels of overnutrition and weight gain during pregnancy have differential effects on fetal growth and organ development. *Reproductive Biology and Endocrinology*.

[41] Kirk SL, Samuelsson AM, Argenton M (2009). Maternal obesity induced by diet in rats permanently influences central processes regulating food intake in offspring. *PLoS ONE*.

[42] Lewis DS, Bertrand HA, McMahan CA, McGill HC, Carey KD, Masoro EJ (1986). Preweaning food intake influences the adiposity of young adult baboons. *Journal of Clinical Investigation*.

[43] McCurdy CE, Bishop JM, Williams SM (2009). Maternal high-fat diet triggers lipotoxicity in the fetal livers of nonhuman primates. *Journal of Clinical Investigation*.

[44] Farley D, Tejero ME, Comuzzie AG (2009). Feto-placental adaptations to maternal obesity in the baboon. *Placenta*.

[45] Grayson BE, Levasseur PR, Williams SM, Smith MS, Marks DL, Grove KL (2010). Changes in melanocortin expression and inflammatory pathways in fetal offspring of nonhuman primates fed a high-fat diet. *Endocrinology*.

[46] Sullivan EL, Grayson B, Takahashi D (2010). Chronic consumption of a high-fat diet during pregnancy causes perturbations in the serotonergic system and increased anxiety-like behavior in nonhuman primate offspring. *Journal of Neuroscience*.

[47] Frias AE, Morgan TK, Evans AE (2011). Maternal high-fat diet disturbs uteroplacental hemodynamics and increases the frequency of stillbirth in a nonhuman primate model of excess nutrition. *Endocrinology*.

[48] Mark PJ, Sisala C, Connor K (2011). A maternal high-fat diet in rat pregnancy reduces growth of the fetus and the placental junctional zone, but not placental labyrinth zone growth. *Journal of the Developmental Origins of Health and Disease*.

[49] Rooney K, Ozanne SE (2011). Maternal over-nutrition and offspring obesity predisposition: targets for preventative interventions. *International Journal of Obesity*.

[50] Bayol SA, Farrington SJ, Stickland NC (2007). A maternal “junk food” diet in pregnancy and lactation promotes an exacerbated taste for “junk food” and a greater propensity for obesity in rat offspring. *The British Journal of Nutrition*.

[51] Nivoit P, Morens C, Van Assche FA (2009). Established diet-induced obesity in female rats leads to offspring hyperphagia, adiposity and insulin resistance. *Diabetologia*.

[52] Ahima RS, Prabakaran D, Flier JS (1998). Postnatal leptin surge and regulation of circadian rhythm of leptin by feeding. Implications for energy homeostasis and neuroendocrine function. *Journal of Clinical Investigation*.

[53] Purcell RH, Sun B, Pass LL, Power ML, Moran TH, Tamashiro KL (2011). Maternal stress and high-fat diet effect on maternal behavior, milk composition, and pup ingestive behavior. *Physiology and Behavior*.

[54] Flint DJ, Travers MT, Barber MC, Binart N, Kelly PA (2005). Diet-induced obesity impairs mammary development and lactogenesis in murine mammary gland. *The American Journal of Physiology—Endocrinology and Metabolism*.

[55] Champagne FA, Weaver ICG, Diorio J, Dymov S, Szyf M, Meaney MJ (2006). Maternal care associated with methylation of the estrogen receptor-alpha1b promoter and estrogen receptor-alpha expression in the medial preoptic area of female offspring. *Endocrinology*.

[56] Meaney MJ, Szyf M (2005). Environmental programming of stress responses through DNA methylation: life at the interface between a dynamic environment and a fixed genome. *Dialogues in Clinical Neuroscience*.

[57] Zhu MJ, Ma Y, Long NM, Du M, Ford SP (2010). Maternal obesity markedly increases placental fatty acid transporter expression and fetal blood triglycerides at midgestation in the ewe. *The American Journal of Physiology—Regulatory Integrative and Comparative Physiology*.

[58] Simar D, Chen H, Lambert K, Mercier J, Morris MJ Interaction between maternal obesity and post-natal over-nutrition on skeletal muscle metabolism.

[59] Bayol SA, MacHaria R, Farrington SJ, Simbi BH, Stickland NC (2009). Evidence that a maternal “junk food” diet during pregnancy and lactation can reduce muscle force in offspring. *European Journal of Nutrition*.

[60] Yan X, Huang Y, Zhao JX (2011). Maternal obesity-impaired insulin signaling in sheep and induced lipid accumulation and fibrosis in skeletal muscle of offspring. *Biology of Reproduction*.

[61] Vickers MH, Breier BH, McCarthy D, Gluckman PD (2003). Sedentary behavior during postnatal life is determined by the prenatal environment and exacerbated by postnatal hypercaloric nutrition. *The American Journal of Physiology—Regulatory Integrative and Comparative Physiology*.

[62] Miles JL, Huber K, Thompson NM, Davison M, Breier BH (2009). Moderate daily exercise activates metabolic flexibility to prevent prenatally induced obesity. *Endocrinology*.

[63] Bellinger L, Sculley DV, Langley-Evans SC (2006). Exposure to undernutrition in fetal life determines fat distribution, locomotor activity and food intake in ageing rats. *International Journal of Obesity*.

[64] Reusens B, Sparre T, Kalbe L (2008). The intrauterine metabolic environment modulates the gene expression pattern in fetal rat islets: prevention by maternal taurine supplementation. *Diabetologia*.

[65] Stoffers DA, Desai BM, DeLeon DD, Simmons RA (2003). Neonatal exendin-4 prevents the development of diabetes in the intrauterine growth retarded rat. *Diabetes*.

[66] Lillycrop KA, Phillips ES, Jackson AA, Hanson MA, Burdge GC (2005). Dietary protein restriction of pregnant rats induces and folic acid supplementation prevents epigenetic modification of hepatic gene expression in the offspring. *Journal of Nutrition*.

[67] Vickers MH, Gluckman PD, Coveny AH (2005). Neonatal leptin treatment reverses developmental programming. *Endocrinology*.

[68] Miles JL, Landon J, Davison M (2009). Prenatally undernourished rats show increased preference for wheel running v. lever pressing for food in a choice task. *The British Journal of Nutrition*.

[69] Zambrano E, Martínez-Samayoa PM, Rodríguez-González GL, Nathanielsz PW (2010). Dietary intervention prior to pregnancy reverses metabolic programming in male offspring of obese rats. *Journal of Physiology*.

[70] Simmons R (2011). Epigenetics and maternal nutrition: nature v. nurture. *Proceedings of the Nutrition Society*.

[71] Gicquel C, El-Osta A, Le Bouc Y (2008). Epigenetic regulation and fetal programming. *Best Practice and Research in Clinical Endocrinology and Metabolism*.

[72] Szyf M (2009). Epigenetics, DNA methylation, and chromatin modifying drugs. *Annual Review of Pharmacology and Toxicology*.

[73] Tang WY, Ho SM (2007). Epigenetic reprogramming and imprinting in origins of disease. *Reviews in Endocrine and Metabolic Disorders*.

[74] Jirtle RL, Skinner MK (2007). Environmental epigenomics and disease susceptibility. *Nature Reviews Genetics*.

[75] Lillycrop KA, Slater-Jefferies JL, Hanson MA, Godfrey KM, Jackson AA, Burdge GC (2007). Induction of altered epigenetic regulation of the hepatic glucocorticoid receptor in the offspring of rats fed a protein-restricted diet during pregnancy suggests that reduced DNA methyltransferase-1 expression is involved in impaired DNA methylation and changes in histone modifications. *The British Journal of Nutrition*.

[76] Sinclair KD, Allegrucci C, Singh R (2007). DNA methylation, insulin resistance, and blood pressure in offspring determined by maternal periconceptional B vitamin and methionine status. *Proceedings of the National Academy of Sciences of the United States of America*.

[77] Weaver IC, Diorio J, Seckl JR, Szyf M, Meaney MJ (2004). Early environmental regulation of hippocampal glucocorticoid receptor gene expression: characterization of intracellular mediators and potential genomic target sites. *Annals of the New York Academy of Sciences*.

[78] Weaver IC, Cervoni N, Champagne FA (2004). Epigenetic programming by maternal behavior. *Nature Neuroscience*.

[79] Doherty AS, Mann MR, Tremblay KD, Bartolomei MS, Schultz RM (2000). Differential effects of culture on imprinted H19 expression in the preimplantation mouse embryo. *Biology of Reproduction*.

[80] Iliopoulos D, Malizos KN, Tsezou A (2007). Epigenetic regulation of leptin affects MMP-13 expression in osteoarthritic chondrocytes: possible molecular target for osteoarthritis therapeutic intervention. *Annals of the Rheumatic Diseases*.

[81] Melzner I, Scott V, Dorsch K (2002). Leptin gene expression in human preadipocytes is switched on by maturation-induced demethylation of distinct CpGs in its proximal promoter. *Journal of Biological Chemistry*.

[82] Stöger R (2006). In vivo methylation patterns of the leptin promoter in human and mouse. *Epigenetics*.

[83] Bouchard L, Thibault S, Guay SP (2010). Leptin gene epigenetic adaptation to impaired glucose metabolism during pregnancy. *Diabetes Care*.

[84] Yokomori N, Tawata M, Onaya T (2002). DNA demethylation modulates mouse leptin promoter activity during the differentiation of 3T3-L1 cells. *Diabetologia*.

[85] Yokomori N, Tawata M, Onaya T (1999). DNA demethylation during the differentiation of 3T3-L1 cells affects the expression of the mouse GLUT4 gene. *Diabetes*.

[86] Carretero MV, Torres L, Latasa U (1998). Transformed but not normal hepatocytes express UCP2. *FEBS Letters*.

[87] Ho SM, Tang WY (2007). Techniques used in studies of epigenome dysregulation due to aberrant DNA methylation: an emphasis on fetal-based adult diseases. *Reproductive Toxicology*.

[88] Godfrey KM, Sheppard A, Gluckman PD (2011). Epigenetic gene promoter methylation at birth is associated with child's later adiposity. *Diabetes*.

[89] Ng SF, Lin RC, Laybutt DR, Barres R, Owens JA, Morris MJ (2010). Chronic high-fat diet in fathers programs beta-cell dysfunction in female rat offspring. *Nature*.

[90] Yazbek SN, Spiezio SH, Nadeau JH, Buchner DA (2010). Ancestral paternal genotype controls body weight and food intake for multiple generations. *Human Molecular Genetics*.

[91] Dunn GA, Bale TL (2011). Maternal high-fat diet effects on third-generation female body size via the paternal lineage. *Endocrinology*.

[92] Poston L, Harthoorn LF, Van Der Beek EM (2011). Obesity in pregnancy: implications for the mother and lifelong health of the child. A consensus statement. *Pediatric Research*.

